# Supplementary material for: Characterization of the AcrIIC1 anti‒CRISPR protein for Cas9‒based genome engineering in E. coli
Source: Commun Biol. 2023 Oct 13;6:1042. doi: 10.1038/s42003-023-05418-5 (PMC10576004; doi:10.1038/s42003-023-05418-5)
Supplement: Supplementary file 2 — Supplementary information [file 42003_2023_5418_MOESM2_ESM.pdf]

Supplementary Table 1 Strains used in this study.

Strain	Description	Plasmid	Antibiotic resistance	Reference
E. coli DH5α	-	-	-	Lab stock
E. coli DH10b	-	-	-	NEB (#C3019H)
E. coli_gfp	Promoter lacUV5 and gfp gene integrated in the genome of E. coli DH10b	-	-	Lab stock
E. coli_gfp:pKD46	Promoter lacUV5 and gfp gene integrated in the genome of E. coli DH10b	pKD46	Ampicillin	This study
E. coli_gfp:pAcr	Promoter lacUV5 and gfp gene integrated in the genome of E. coli DH10b	pAcr	Ampicillin	This study

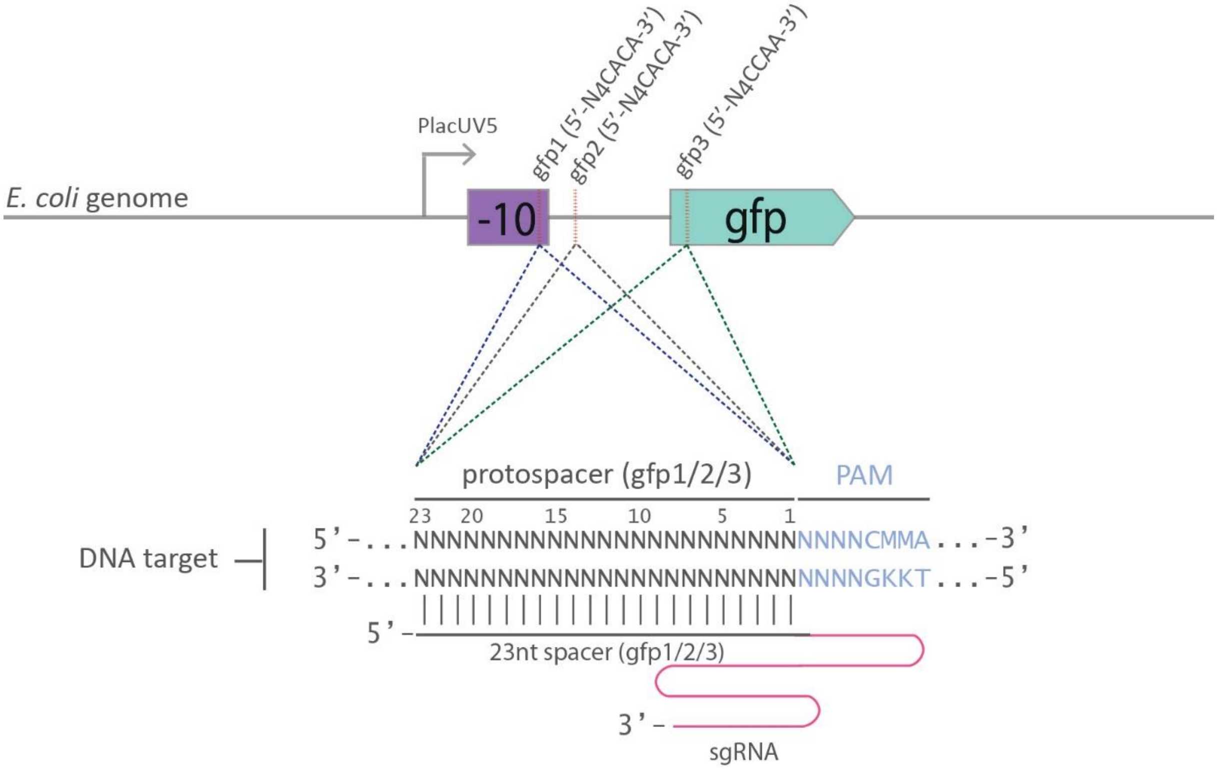

Supplementary Figure 1 Schematic illustration of the genomic location of the targeted protospacers (gfp1/2/3). Protospacers gfp1 and gfp2 are located in the lacUV5 promoter of the *gfp* gene and are flanked by the 5'-N_4CACA-3' PAM, while protospacer gfp3 is found in the beginning of the *gfp* gene and is flanked by the 5'-N_4CCAA-3' PAM. Red dashed lines denote the position of each protospacer in the *E. coli* genome, while the purple square represents the -10 box of P_{lacUV5}.

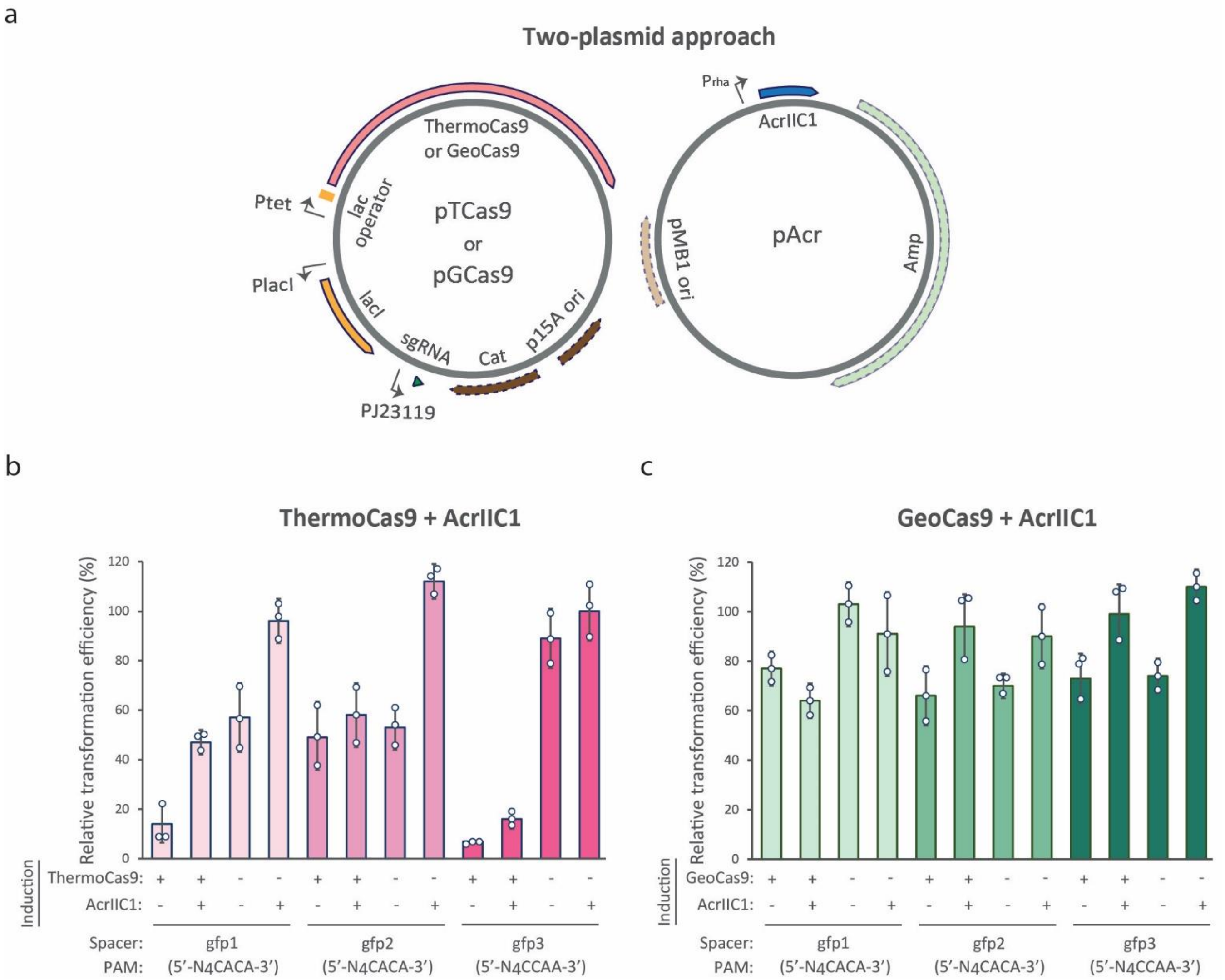

Supplementary Figure 2 Two-plasmid approach for AcrIIC1-mediated inhibition of ThermoCas9 and GeoCas9 *in vivo* cleavage activity. a Schematic illustration of the constructs transformed into the *E. coli_gfp* strain in killing-inhibition assays. **b, c** Transformation efficiency of *E. coli_gfp* cells that express AcrIIC1 for inhibition of either ThermoCas9- (**b**) or GeoCas9- (**c**) based cleavage activity. The plus (+) and minus (-) symbols represent induction (1000 μM IPTG for Cas9; 0.2% L-rhamnose for AcrIIC1) and absence of induction of protein expression, respectively. Bar graphs were created based on results from three independent biological replicates shown as circles. Error bars represent the standard deviation.

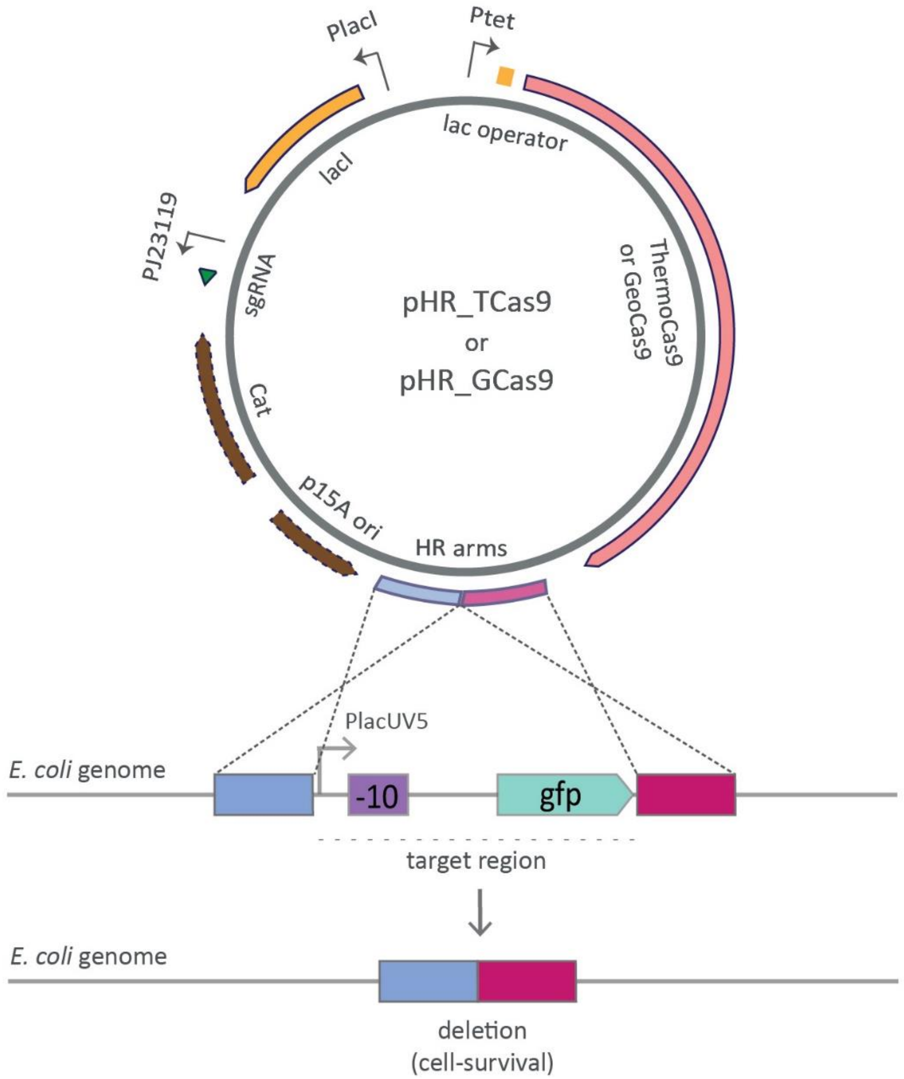

Supplementary Figure 3 ThermoCas9- and GeoCas9-based genome engineering in *E. coli*. Schematic illustration of the pHRs_TCas9 and pHRs_GCas9 constructs for ThermoCas9- and GeoCas9-based genome editing in *E. coli_gfp* that carries the λ-red recombineering-expressing pKD46 plasmid. Homologous recombination arms (~600 bp each) of the target region were introduced to the previously constructed targeting plasmids to allow for λ-red-mediated deletion of the genomic *gfp* gene and along with the P_{lacUV5}, and subsequent counter-selection of the non-edited cells. Six spacers for each nuclease, designed to target protospacers within the sequence of the *gfp* gene and flanked by PAMs of variable preference, were inserted in the 5' end of the sgRNA-expressing module.

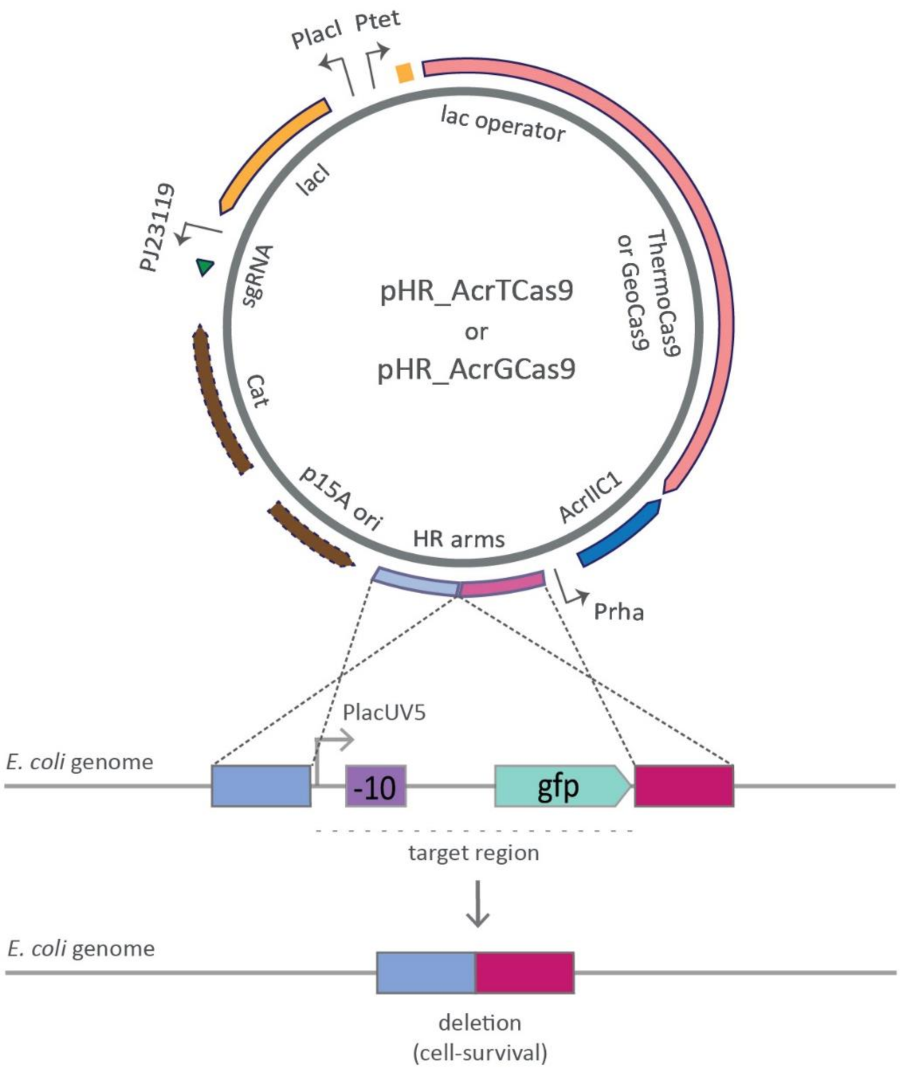

Supplementary Figure 4 AcrIIC1-mediated inhibition of ThermoCas9- and GeoCas9-based genome engineering in *E. coli*. Schematic illustration of the pHRs_AcrTCas9 and pHRs_AcrGCas9 constructs for AcrIIC1-mediated inhibition of ThermoCas9- and GeoCas9-based genome editing in *E. coli_gfp* that carries the λ-red recombineering-expressing pKD46 plasmid. The AcrIIC1 expressing module was inserted into the previously constructed editing plasmids to block Cas9-based counter-selection of non-edited cells.

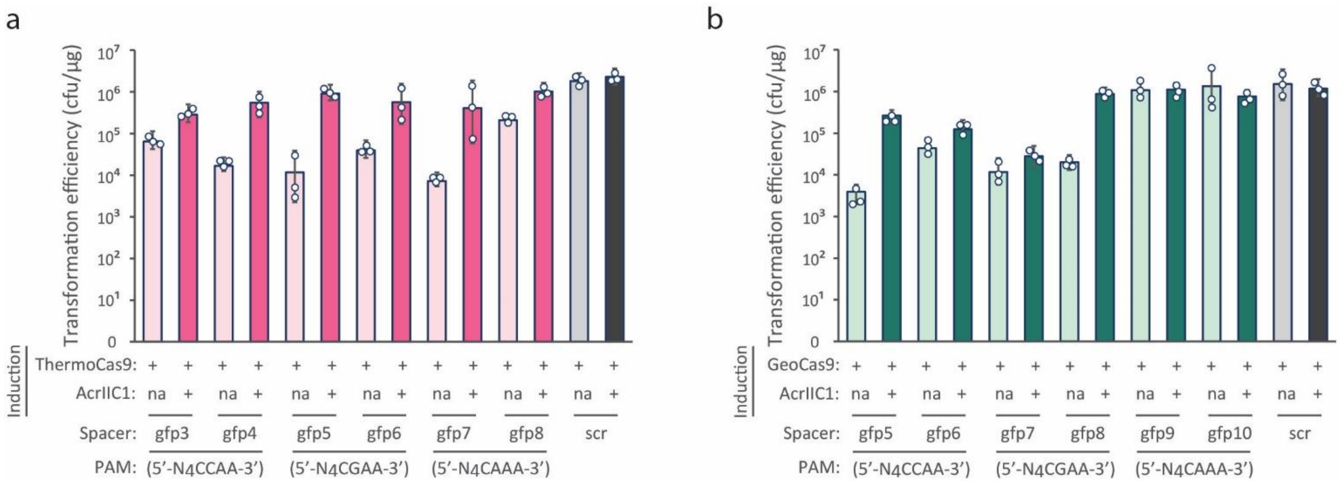

Supplementary Figure 5 AcrIIC1-mediated inhibition of ThermoCas9- and GeoCas9-based cell killing during genome engineering in *E. coli*. Transformation efficiency of *E. coli_gfp* cells that express either ThermoCas9 (**a**) or GeoCas9 (**b**), guided by sgRNA with spacers gfp3-gfp8 and gfp5-gfp10, respectively. Light-coloured bars indicate full expression of Cas9 and absence of the *acriic1* gene ('not applicable' AcrIIC1 expression induction, 'na') in the genome editing plasmid, while dark-coloured bars indicate full expression of both Cas9 and AcrIIC1. Bar graphs were created based on results from three independent biological replicates shown as circles. Error bars represent the standard deviation.

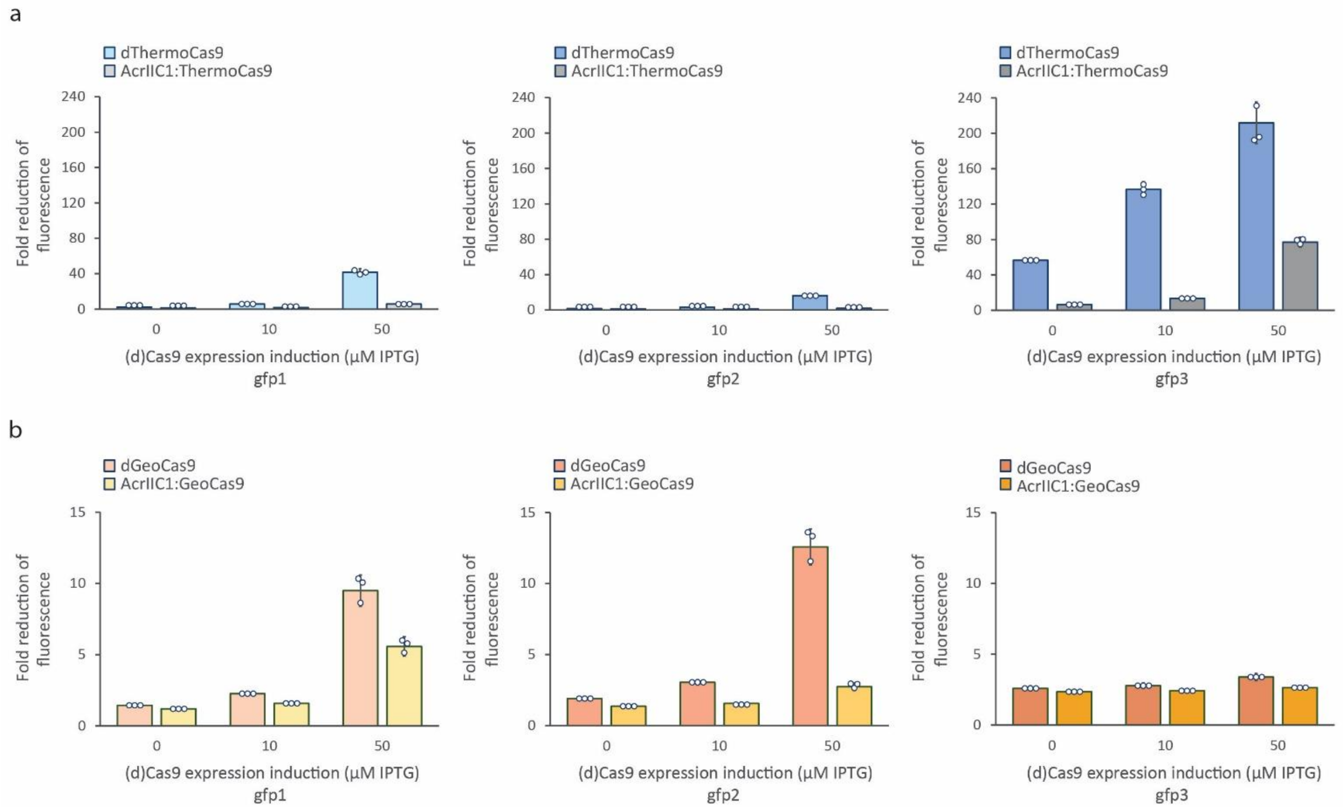

Supplementary Figure 6 Comparison of dThermoCas9 and dGeoCas9 with their AcrIIC1:Cas9 complexes for silencing of the genome-integrated *gfp* gene in *E. coli*. Flow cytometry-based fluorescence loss assays show fold reduction of fluorescence of *E. coli_gfp* cells that express (**a**) dThermoCas9 (blue) or AcrIIC1:ThermoCas9 (grey); (**b**) dGeoCas9 (orange) or AcrIIC1:GeoCas9 (yellow), guided by sgRNA with spacers gfp1, gfp2, or gfp3. These spacers correspond to protospacers flanked by certain PAMs (5'-N4CACA-3' for gfp1 and gfp2; 5'-N4CCAA-3' for gfp3). The expression of the (d)Cas9 proteins was induced using variable IPTG concentrations (0, 10, 50 μM), while the expression of AcrIIC1 was always induced with 0.2% L-rhamnose in this two-plasmid approach. Bar graphs were created based on results from three independent biological replicates shown as circles. Error bars represent the standard deviation.

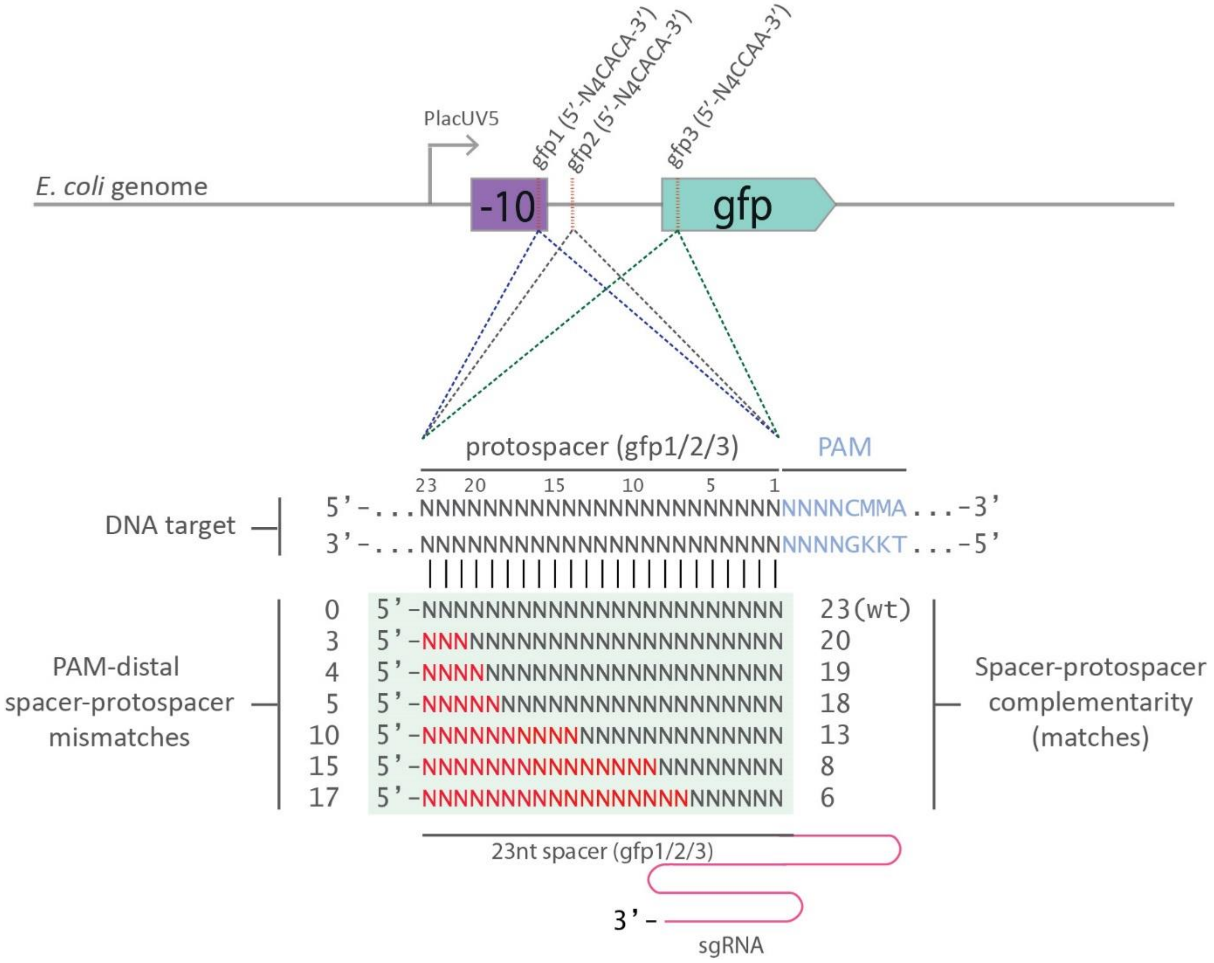

Supplementary Figure 7 Spacer-protospacer mismatch library for examination of the targeting and binding specificity of ThermoCas9, GeoCas9, their deactivated variants, and their AcrIIC1:Cas9 complexes in *E. coli_gfp*. Multiple consecutive mismatches (red) were introduced in the 5'-end of the spacer (gfp1, gfp2, or gfp3), while the rest of the nucleotides of the spacer remained matched (black) to the corresponding protospacer. The PAM is shown in light blue, and the sgRNA-expressing module is depicted in pink.

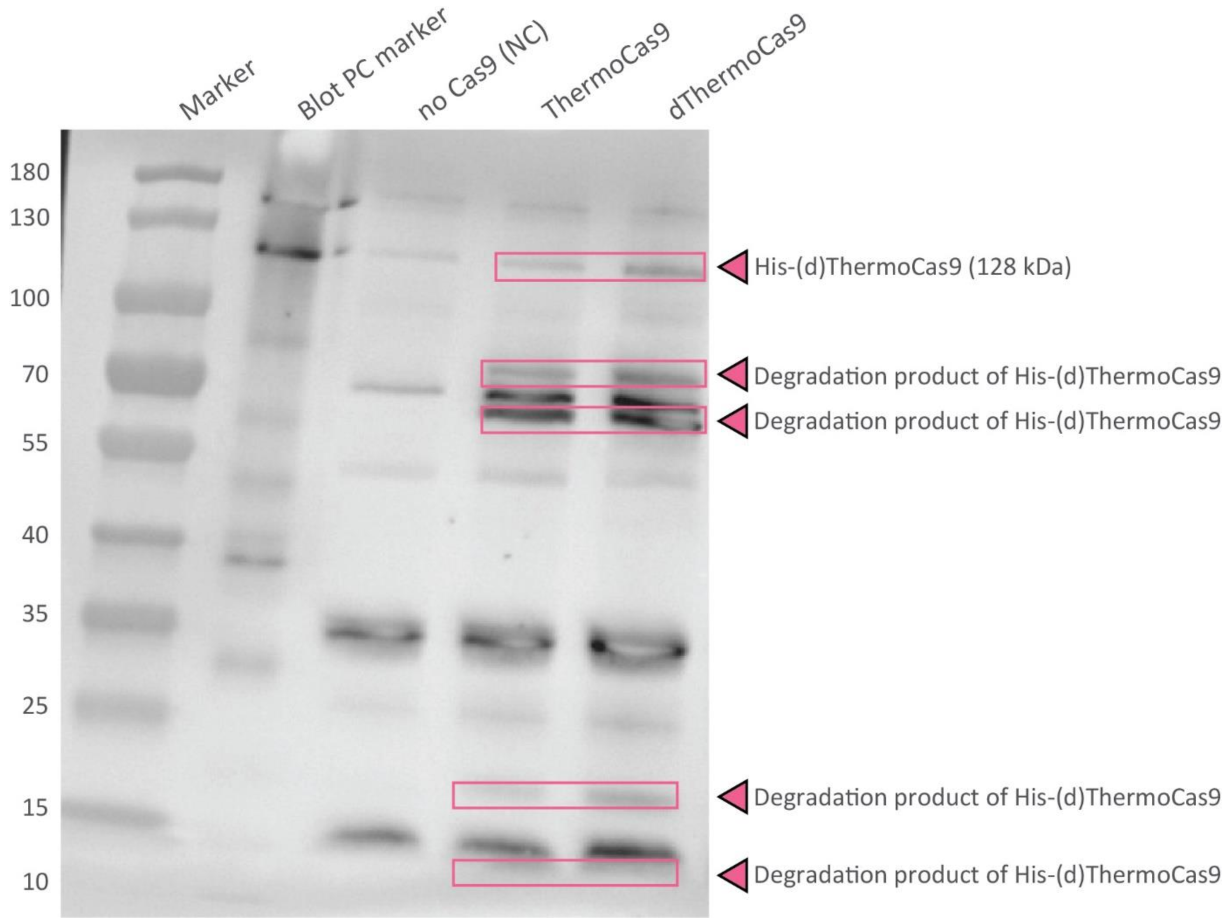

Supplementary Figure 8 Comparison of His-ThermoCas9 and His-dThermoCas9 expression in *E. coli_gfp*. Western-blot assay shows the expression of His6-ThermoCas9, His6-dThermoCas9, and their degradation products (pink boxes). A band of approximately 128 kDa is observed not only in the presence (His-ThermoCas9, His-dThermoCas9) but also in the absence (no His-(d)ThermoCas9; NC) of His-(d)ThermoCas9. So, *E. coli_gfp* cells express a protein of the same size as the His-(d)ThermoCas9, not allowing us to draw any conclusions. However, His-(d)ThermoCas9 degradation products are present in the His-(d)ThermoCas9 samples, while they are absent in the no His-(d)ThermoCas9 sample. The intensity of these bands is comparable between the His-ThermoCas9 and the His-(d)ThermoCas9 samples.

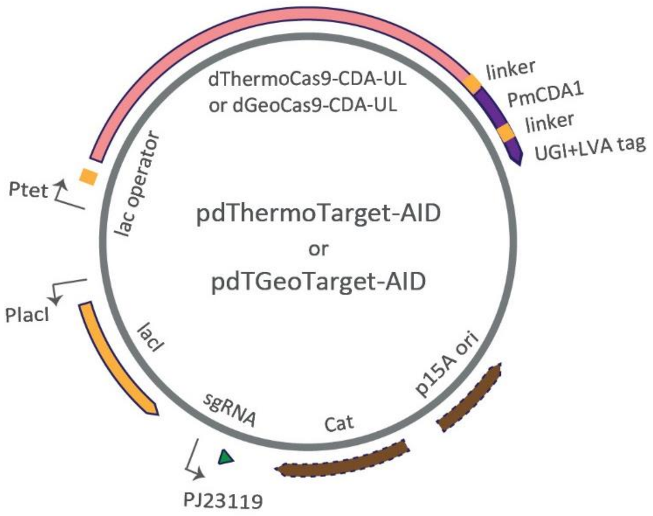

Supplementary Figure 9 Base-editing in *E. coli* using dThermoCas9, dGeoCas9, and their AcrIIC1:Cas9 complexes. Schematic illustration of the dCas9 and AcrIIC1:Cas9 base-editing constructs. The *pmcda1* and *ugi* genes were fused to the 3' end of the *dcas9* or active *cas9* genes of the previously described pdTCas9, pdGCas9, pAcrTCas9, and pAcrGCas9 plasmids to enable base-editing. For each base-editor, six spacers targeting the *gfp* gene of the *E. coli_gfp* genome were applied.

a

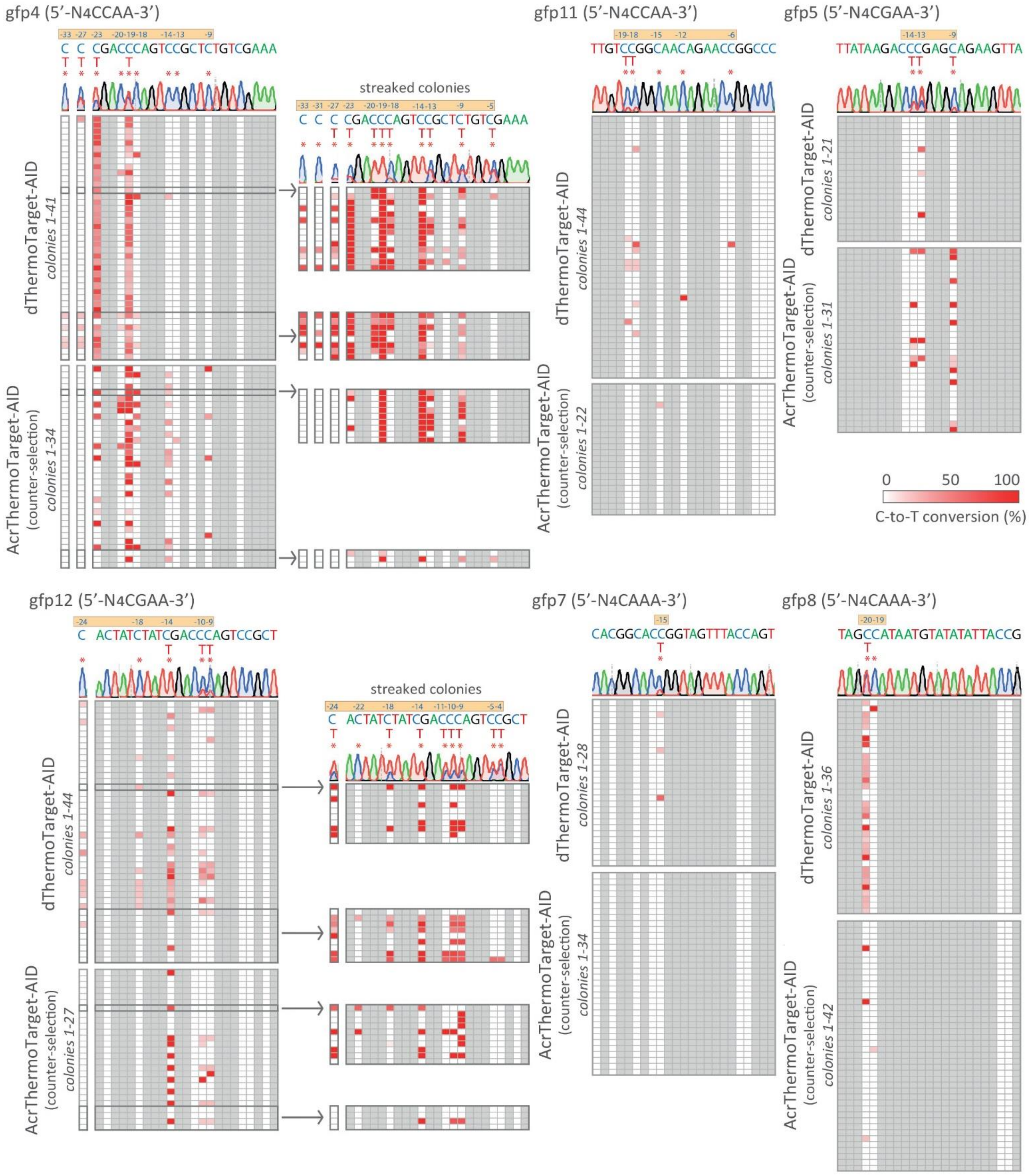
gfp4 (5′-N4CCAA-3′)
-33 -27 -23 -20-19-18 -14-13 -9
CCCGACCCAGTCCGCTCTGTCGAAA
streaked colonies
-33 -31 -27 -23 -20-19-18 -14-13 -9 -5
CCCCGACCCAGTCCGCTCTGTCGAAA
dThermoTarget-AID
colonies 1-41
AcrThermoTarget-AID
(counter-selection)
colonies 1-34
gfp11 (5′-N4CCAA-3′)
-19-18 -15 -12 -6
TTGTCCGGCAACAGAACCGGCCC
dThermoTarget-AID
colonies 1-44
AcrThermoTarget-AID
(counter-selection)
colonies 1-22
gfp5 (5′-N4CGAA-3′)
-14-13 -9
TTATAAGACCCGAGCAGAAGTTA
dThermoTarget-AID
colonies 1-21
AcrThermoTarget-AID
(counter-selection)
colonies 1-31
0 50 100
C-to-T conversion (%)
gfp12 (5′-N4CGAA-3′)
-24 -18 -14 -10-9
C ACTATCTATCGACCCAGTCCGCT
streaked colonies
-24 -22 -18 -14 -11-10-9 -5-4
C ACTATCTATCGACCCAGTCCGCT
dThermoTarget-AID
colonies 1-44
AcrThermoTarget-AID
(counter-selection)
colonies 1-27
gfp7 (5′-N4CAAA-3′)
-15
CACGGCACCGGTAGTTTACCAGT
dThermoTarget-AID
colonies 1-28
AcrThermoTarget-AID
(counter-selection)
colonies 1-34
gfp8 (5′-N4CAAA-3′)
-20-19
TAGCCATAATGTATATATTACCG
dThermoTarget-AID
colonies 1-36
AcrThermoTarget-AID
(counter-selection)
colonies 1-42

b

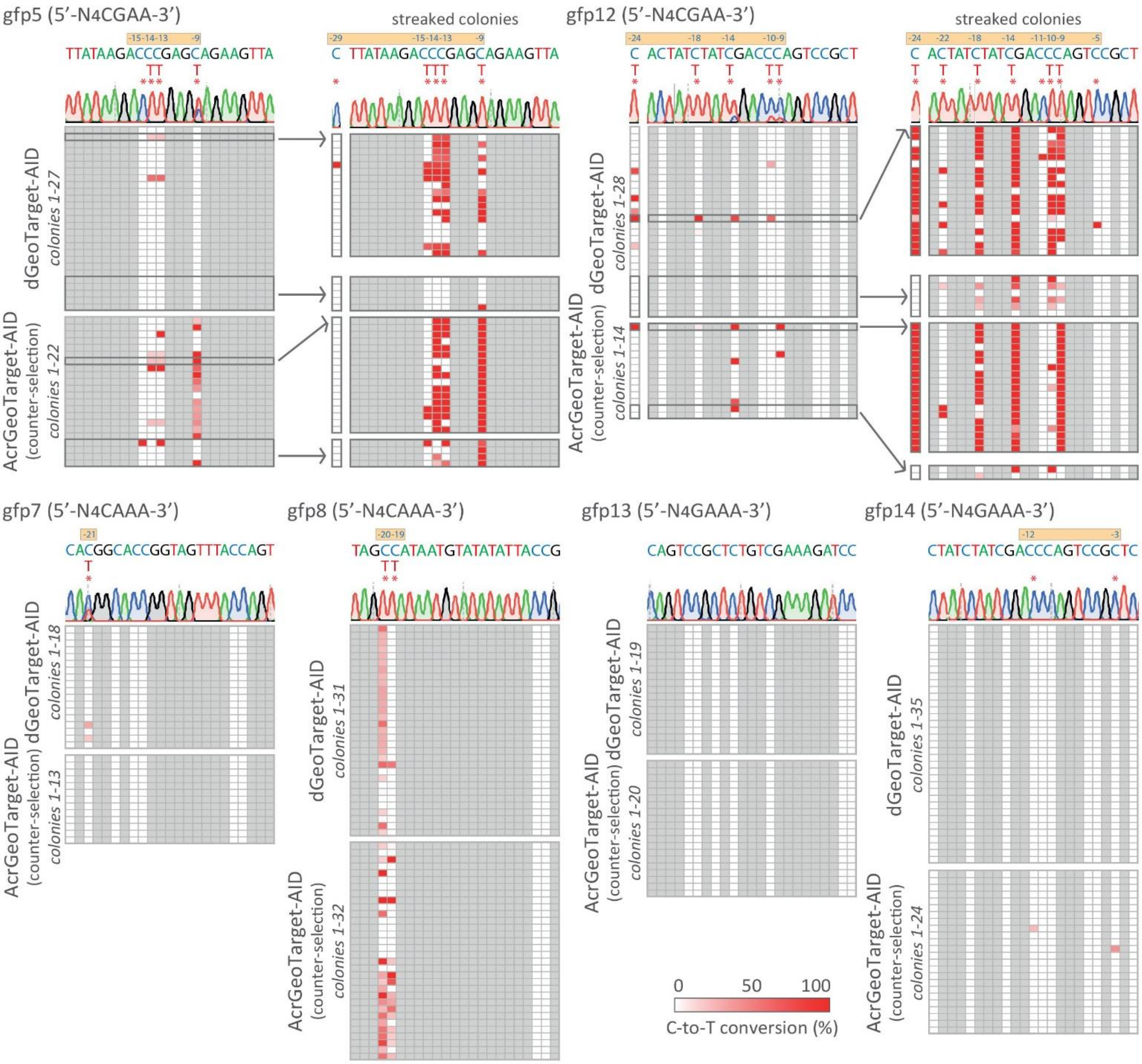

Supplementary Figure 10 Characterisation of the dCas9 and AcrIIC1:Cas9 base-editors in *E. coli*. (a) ThermoTarget-AID, or **(b)** GeoTarget-AID editing heatmaps depicting the percentage of C•G to T•A conversion in every C-position within or immediately upstream of protospacers (x axis) located in the genomic *gfp* gene of *E. coli_gfp* single colonies transformed with the base-editing vectors (y axis). The percentages resulted from high-throughput Sanger sequencing analysis of several single colonies, employing a variation of the on-line tool 'EditR' and setting as threshold p ≤ 0.5. White boxes represent no base-editing, light to darker pink boxes represent increasing base-editing efficiencies, and red boxes represent 100% base-editing efficiency. The red asterisks indicate edited Cs at a certain position in at least one of the screened colonies. The yellow boxes indicate the positions of edited Cs reported for the screened colonies. For each protospacer, the Sanger sequencing chromatogram of a random, screened single colony is presented as an example.

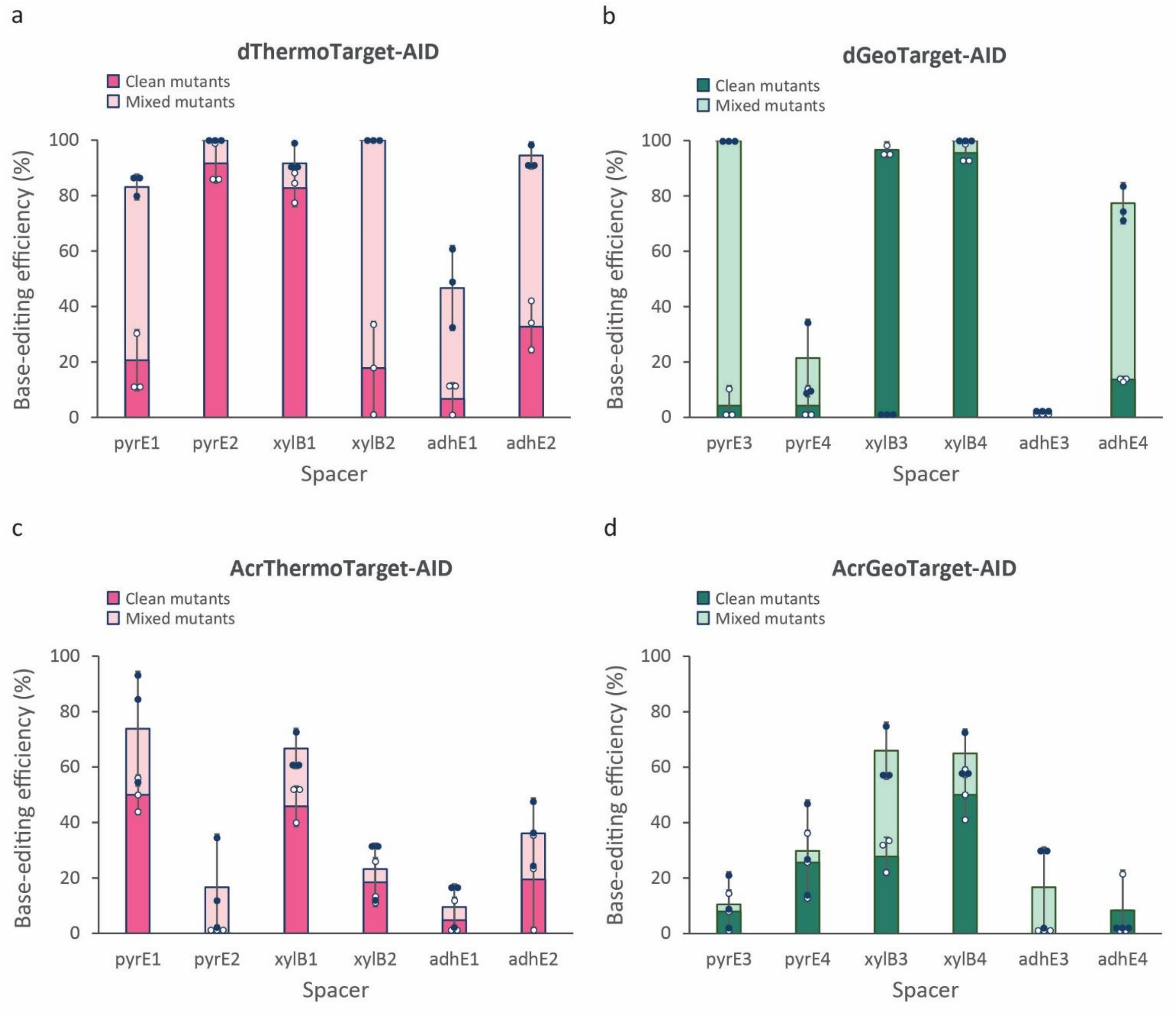

Supplementary Figure 11 Base-editing at endogenous sites in *E. coli* using type II-C cytidine base-editors and their AcrIIC1:Cas9 complexes. Base-editing efficiency (%) of the dThermoCas9 (**a**), dGeoCas9 (**b**), AcrIIC1:ThermoCas9 (**c**), and AcrIIC1:GeoCas9 (**d**) base-editors in *E. coli_gfp*. The base-editing efficiency (%) represents the % of the number of edited versus screened single colonies, while the clean-mutant efficiency (%) represents the % of clean-mutant versus screened single colonies. Dark pink and green bars indicate clean mutant editing efficiencies, while light pink and green bars represent mixed mutant editing efficiencies. All protospacers were flanked by an optimal PAM (5’-N4CCAA-3’ for the ThermoCas9 base-editors; 5’-N4CGAA-3’ for the GeoCas9 base-editors). Bar graphs were created based on results from three independent biological replicates shown as white circles for clean mutants and as black circles for mixed mutants. Error bars represent the standard deviation.

a

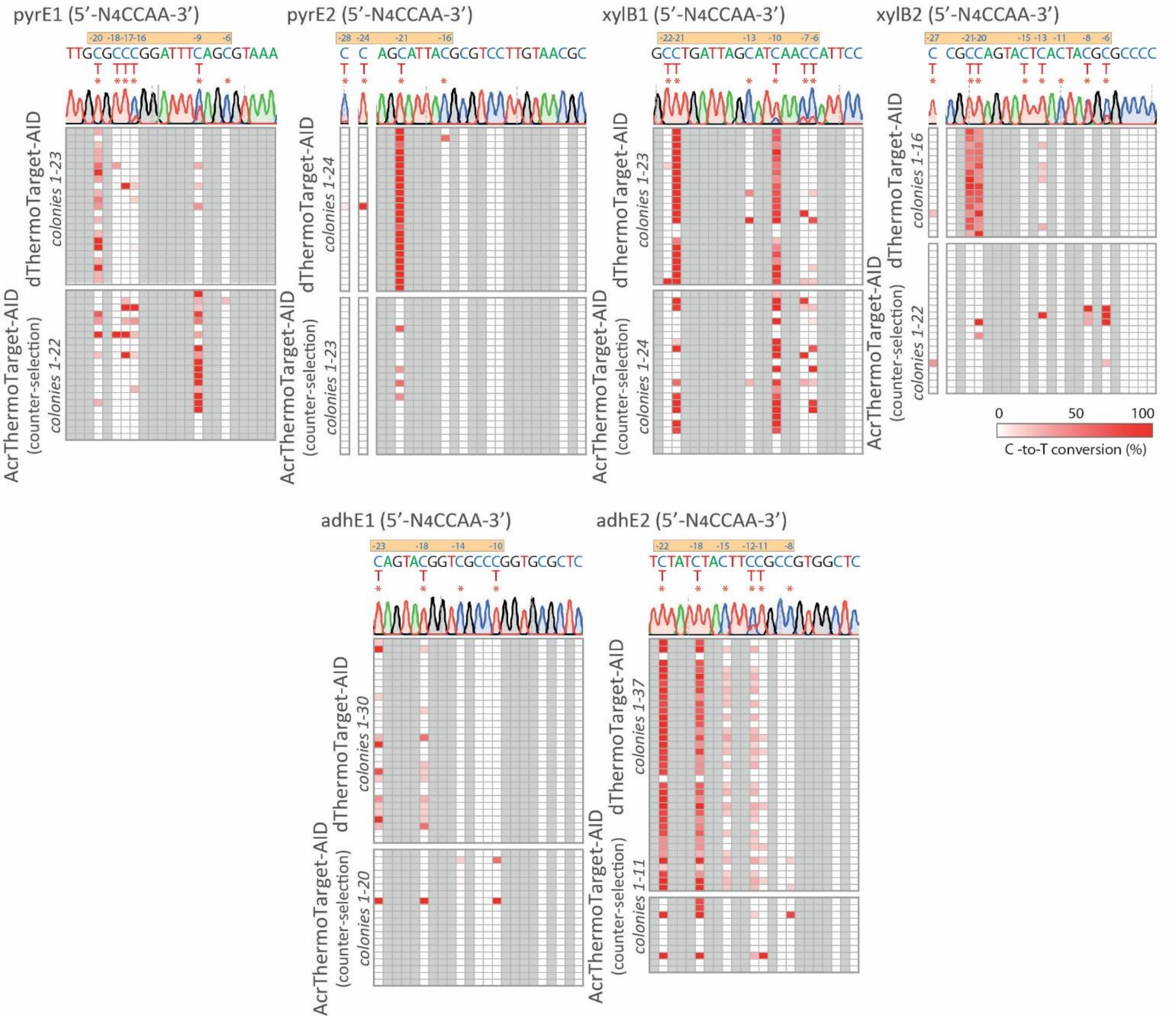
pyrE1 (5′-N4CCAA-3′)
pyrE2 (5′-N4CCAA-3′)
xylB1 (5′-N4CCAA-3′)
xylB2 (5′-N4CCAA-3′)
adhE1 (5′-N4CCAA-3′)
adhE2 (5′-N4CCAA-3′)
dThermoTarget-AID
AcrThermoTarget-AID
(counter-selection)
colonies 1-23
colonies 1-22
colonies 1-24
colonies 1-16
colonies 1-30
colonies 1-20
colonies 1-37
colonies 1-11
0
50
100
C -to-T conversion (%)

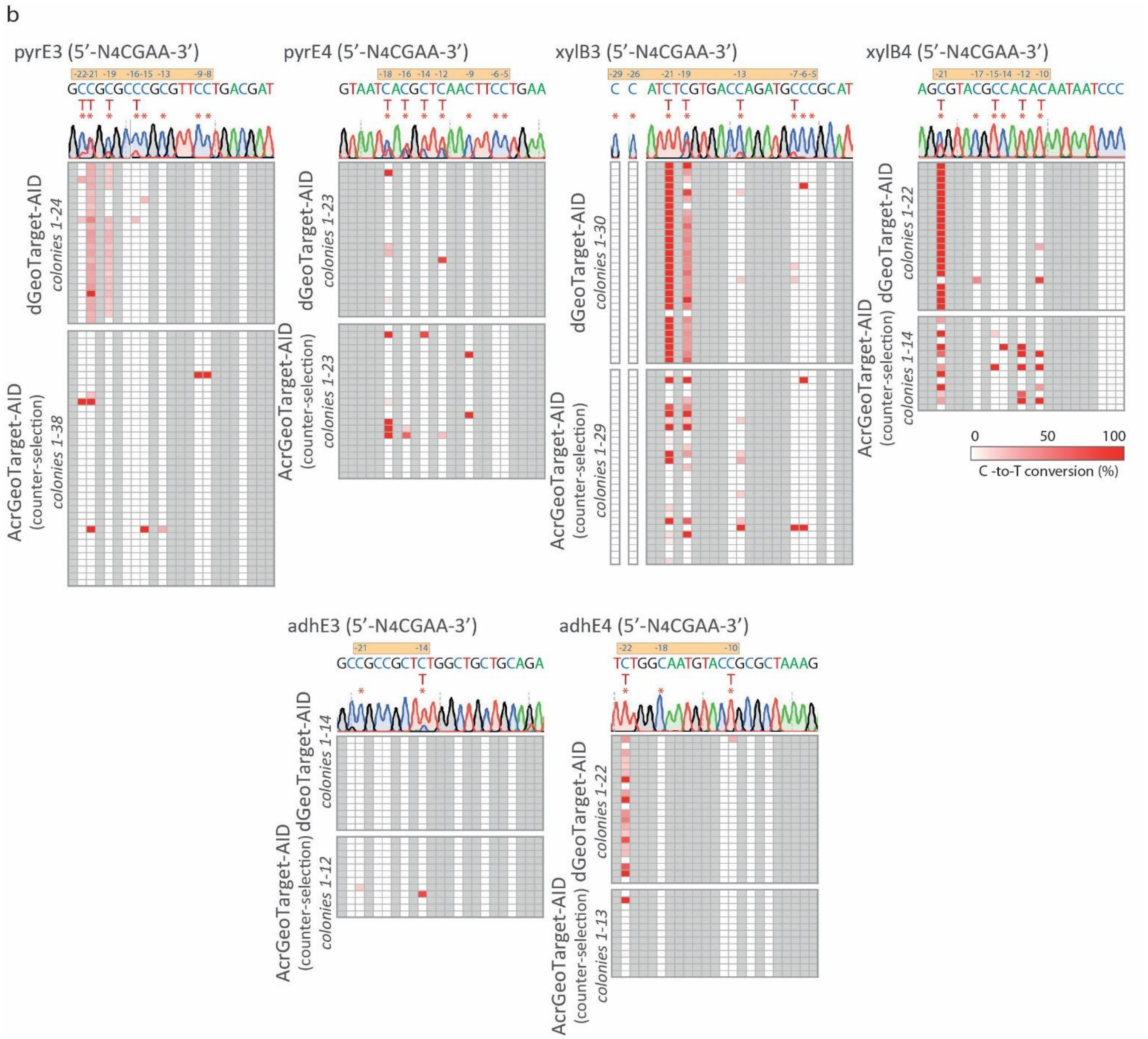

Supplementary Figure 12 Characterisation of the dCas9 and AcrIIC1:Cas9 base-editors at endogenous sites in *E. coli*. (a) ThermoTarget-AID, or **(b)** GeoTarget-AID editing heatmaps depicting the percentage of C•G to T•A conversion in every C-position within or immediately upstream of protospacers (x axis) located in the genomic *pyrE*, *xylB* and *adhE* genes of *E. coli_gfp* single colonies transformed with the base-editing vectors (y axis). The percentages resulted from high-throughput Sanger sequencing analysis of several single colonies, employing a variation of the on-line tool 'EditR' and setting as threshold p ≤ 0.5. White boxes represent no base-editing, light to darker pink boxes represent increasing base-editing efficiencies, and red boxes represent 100% base-editing efficiency. The red asterisks indicate edited Cs at a certain position in at least one of the screened colonies. The yellow boxes indicate the positions of edited Cs reported for the screened colonies. For each protospacer, the Sanger sequencing chromatogram of a random, screened single colony is presented as an example.
