# Supplementary material for: Characterization of the AcrIIC1 anti‒CRISPR protein for Cas9‒based genome engineering in E. coli
Source: Commun Biol. 2023 Oct 13;6:1042. doi: 10.1038/s42003-023-05418-5 (PMC10576004; doi:10.1038/s42003-023-05418-5)
Supplement: Supplementary file 3 — Description of Additional Supplementary Data [file 42003_2023_5418_MOESM3_ESM.docx]

**Description of Additional Supplementary Files**

**File name:** Supplementary Data 1

**Description:** Plasmids used in this study.

**File name:** Supplementary Data 2

**Description:** Spacers used in this study.

**File name:** Supplementary Data 3

**Description:** Primers/Oligonucleotides used in this study.

**File name:** Supplementary Data 4

**Description:** DNA sequences of (fusion) genes used in this study.

**File name:** Supplementary Data 5

**Description:** Amino acid sequences of (fusion) proteins used in this study..
