# Supplementary material for: Characterization of the AcrIIC1 anti‒CRISPR protein for Cas9‒based genome engineering in E. coli
Source: Commun Biol. 2023 Oct 13;6:1042. doi: 10.1038/s42003-023-05418-5 (PMC10576004; doi:10.1038/s42003-023-05418-5)
Supplement: Supplementary file 5 — Reporting summary [file 42003_2023_5418_MOESM5_ESM.pdf]

nature portfolio

Corresponding author(s): Raymond H.J. Staals; Ioannis Mougiakos

Last updated by author(s): Aug 13, 2023

Reporting Summary

Nature Portfolio wishes to improve the reproducibility of the work that we publish. This form provides structure for consistency and transparency in reporting. For further information on Nature Portfolio policies, see our Editorial Policies and the Editorial Policy Checklist.

Statistics

For all statistical analyses, confirm that the following items are present in the figure legend, table legend, main text, or Methods section.

n/a	Confirmed	
☐	☒	The exact sample size (*n*) for each experimental group/condition, given as a discrete number and unit of measurement
☐	☒	A statement on whether measurements were taken from distinct samples or whether the same sample was measured repeatedly
☐	☒	The statistical test(s) used AND whether they are one- or two-sided<br>*Only common tests should be described solely by name; describe more complex techniques in the Methods section.*
☐	☒	A description of all covariates tested
☐	☒	A description of any assumptions or corrections, such as tests of normality and adjustment for multiple comparisons
☐	☒	A full description of the statistical parameters including central tendency (e.g. means) or other basic estimates (e.g. regression coefficient) AND variation (e.g. standard deviation) or associated estimates of uncertainty (e.g. confidence intervals)
☐	☒	For null hypothesis testing, the test statistic (e.g. *F*, *t*, *r*) with confidence intervals, effect sizes, degrees of freedom and *P* value noted<br>*Give P values as exact values whenever suitable.*
☒	☐	For Bayesian analysis, information on the choice of priors and Markov chain Monte Carlo settings
☒	☐	For hierarchical and complex designs, identification of the appropriate level for tests and full reporting of outcomes
☒	☐	Estimates of effect sizes (e.g. Cohen's *d*, Pearson's *r*), indicating how they were calculated

Our web collection on statistics for biologists contains articles on many of the points above.

Software and code

Policy information about availability of computer code

Data collection	The AttuneTM Cytometric Software v5.2.0. was used for flow cytometry data collection.
Data analysis	The free on-line tool EditR was used for analysis of Sanger sequencing results in base-editing assays. The AttuneTM Cytometric Software v5.2.0. and Microsoft Excel were used for the analysis of flow cytometry results. All bar-graphs were created in Microsoft Excel. All figures were subjected to Adobe Illustrator 2023.

For manuscripts utilizing custom algorithms or software that are central to the research but not yet described in published literature, software must be made available to editors and reviewers. We strongly encourage code deposition in a community repository (e.g. GitHub). See the Nature Portfolio guidelines for submitting code & software for further information.

Data

Policy information about availability of data

All manuscripts must include a data availability statement. This statement should provide the following information, where applicable:

- Accession codes, unique identifiers, or web links for publicly available datasets
- A description of any restrictions on data availability
- For clinical datasets or third party data, please ensure that the statement adheres to our policy

All data supporting the findings of this study are available within the paper and its Supplementary Information/Data. Plasmid maps have been deposited in Addgene and are publicly available using the following Addgene names and IDs: pTCas9_scr (#207566), pGCas9_scr (#207567), pdTCas9_scr (#207568), pdGCas9_scr

(#207569), pAcr (#207570), pAcrTCas9_scr (#207571), pAcrGCas9_scr (#207572), pdThermoTarget-AID_scr (#207573), pdGeoTarget-AID_gfp5 (#207574), pAcrThermoTarget-AID_scr (#207575) and pAcrGeoTarget-AID_gfp5 (#207576). Any additional data are available from the corresponding author on reasonable request.

Human research participants

Policy information about studies involving human research participants and Sex and Gender in Research.

Reporting on sex and gender	*Use the terms sex (biological attribute) and gender (shaped by social and cultural circumstances) carefully in order to avoid confusing both terms. Indicate if findings apply to only one sex or gender; describe whether sex and gender were considered in study design whether sex and/or gender was determined based on self-reporting or assigned and methods used. Provide in the source data disaggregated sex and gender data where this information has been collected, and consent has been obtained for sharing of individual-level data; provide overall numbers in this Reporting Summary. Please state if this information has not been collected. Report sex- and gender-based analyses where performed, justify reasons for lack of sex- and gender-based analysis.*
Population characteristics	*Describe the covariate-relevant population characteristics of the human research participants (e.g. age, genotypic information, past and current diagnosis and treatment categories). If you filled out the behavioural & social sciences study design questions and have nothing to add here, write "See above."*
Recruitment	*Describe how participants were recruited. Outline any potential self-selection bias or other biases that may be present and how these are likely to impact results.*
Ethics oversight	*Identify the organization(s) that approved the study protocol.*

Note that full information on the approval of the study protocol must also be provided in the manuscript.

Field-specific reporting

Please select the one below that is the best fit for your research. If you are not sure, read the appropriate sections before making your selection.

☒ Life sciences ☐ Behavioural & social sciences ☐ Ecological, evolutionary & environmental sciences

For a reference copy of the document with all sections, see nature.com/documents/nr-reporting-summary-flat.pdf

Life sciences study design

All studies must disclose on these points even when the disclosure is negative.

Sample size	Sample size determination was not relevant is this study.
Data exclusions	No data was excluded.
Replication	At least three independent biological replicates have been performed in all assays and are indicated.
Randomization	Randomization was not relevant is this study because there was no null hypothesis to statistically test for.
Blinding	Blinding was not relevant in this study because there was no null hypothesis to statistically test for.

Reporting for specific materials, systems and methods

We require information from authors about some types of materials, experimental systems and methods used in many studies. Here, indicate whether each material, system or method listed is relevant to your study. If you are not sure if a list item applies to your research, read the appropriate section before selecting a response.

Materials & experimental systems

n/a	Involved in the study	
☒	☐	Antibodies
☒	☐	Eukaryotic cell lines
☒	☐	Palaeontology and archaeology
☒	☐	Animals and other organisms
☒	☐	Clinical data
☒	☐	Dual use research of concern

Methods

n/a	Involved in the study	
☒	☐	ChIP-seq
☐	☒	Flow cytometry
☒	☐	MRI-based neuroimaging

Flow Cytometry

Plots

Confirm that:

- [x] The axis labels state the marker and fluorochrome used (e.g. CD4-FITC).
- [x] The axis scales are clearly visible. Include numbers along axes only for bottom left plot of group (a 'group' is an analysis of identical markers).
- [x] All plots are contour plots with outliers or pseudocolor plots.
- [x] A numerical value for number of cells or percentage (with statistics) is provided.

Methodology

Sample preparation	The sample preparation is extensively described in the section 'Binding assays' of Methods.
Instrument	Attune NxT Flow Cytometer device.
Software	AttuneTM Cytometric Software v5.2.0.
Cell population abundance	We examined the GFP intensity 405-512/25 of at least 30,000 single cells per sample.
Gating strategy	Gating of bacterial cells was performed through a polygonal gate centered around 40,000 FSC-A (x-axis) and 150,000 SSC-A (y-axis) within the range of 5,487 - 88,034 FSC-A (x-axis) and 8,126 - 318,570 SSC-A (y-axis). Gating of single cells was performed through a polygonal gate centered around 150,000 SSC-A (x-axis) and 150,000 SSC-H (y-axis) within the range of 7267 - 280762 SSC-A (x-axis) and 8909 - 264113 SSC-H (y-axis). Gating of GFP signal was performed through a one-dimensional gate in the VL2-A channel (405 nm excitation, 512/25 emission), considering all events of 307 GFP VL2-A or higher. Voltage was set to 480 for the VL2-A channel, while for all the rest channels it was set to 400.

- [ ] Tick this box to confirm that a figure exemplifying the gating strategy is provided in the Supplementary Information.

March 2021
